# Environmental *Vibrio cholerae* Strains Harboring Cholera Toxin and *Vibrio* Pathogenicity Island 1, Nigeria, 2008–2015

**DOI:** 10.3201/eid3011.240495

**Published:** 2024-11

**Authors:** Sergio Morgado, Akinsinde Adewale, Iwalokun Abiodun, Salako Lawal, Fernanda Freitas, Érica Fonseca, Ana Carolina Vicente

**Affiliations:** Instituto Oswaldo Cruz, Rio de Janeiro, Brazil (S. Morgado, F. Freitas, É. Fonseca, A.C. Vicente); Nigerian Institute of Medical Research, Yaba, Nigeria (A. Adewale, I. Abiodun, S. Lawai)

**Keywords:** Vibrio cholerae, Vibrio cholerae lineages, virulence, outbreak, diarrhea, human infection, waterborne infection, bacteria, Nigeria, enteric infections, antimicrobial resistance

## Abstract

Analysis of clinical and environmental *Vibrio cholerae* O1 strains obtained during 2008–2015 in Nigeria showed that lineages Afr9 and Afr12 carrying cholera toxin and *Vibrio* pathogenicity island 1 can be isolated from water. Our findings raise concerns about the role of the environment in maintenance and emergence of cholera outbreaks in Nigeria.

Nigeria is one of the current cholera hotspots in Africa (*1*). The World Health Organization report on cholera cases in countries in Africa for January 2022–December 2023 showed that most cases in West Africa were in Nigeria (n = 26,452) (*2*).

In 1970, the seventh cholera pandemic in Africa was initiated by the *Vibrio cholerae* O1 El Tor lineage (7PET), which became endemic to many countries in Africa (*3*). The pathogenicity of that lineage is characterized by 2 factors: cholera toxin, encoded by the *ctx*AB operon in the lysogenic bacteriophage CTXΦ, and the toxin coregulated pilus (TCP), encoded on the *Vibrio* pathogenicity island 1 and an essential factor for intestinal colonization and CTXΦ uptake (*4*). Weill et al. reconstructed the spatiotemporal spread of cholera in Africa during the seventh and current pandemics, showing that the 7PET lineage evolved into >13 sublineages and that the Afr9 and Afr12 lineages are the main sublineages causing cholera outbreaks in Nigeria and Cameroon (West Africa) (*3*). As part of efforts to provide information for cholera control, we used conventional microbiology, whole-genome sequencing, comparative genomics, and phylogenetic analysis to characterize clinical and environmental *V. cholerae* O1 strains obtained during the 2008–2015 cholera outbreaks in Nigeria.

We analyzed 24 *V. cholerae* strains comprising isolates from clinical (n = 16), environmental (n = 5), and unknown (n = 3) sources (Appendix). We used standard culture methods to identify and confirm that all strains were *V. cholerae* serogroup O1. We sequenced the genomes of those strains by using an Illumina Hiseq 2500 (https://www.illumina.com), assembled them with SPAdes v3.15.2 (https://github.com/ablab/spades), and analyzed them with Abricate by using the CARD and VFDB databases (https://github.com/tseemann/abricate). We analyzed the 24 environmental/clinical genomes from our study along with 36 other representative environmental/clinical *V. cholerae* genomes from Africa spanning all Afr sublineages (Afr1–12) of the seventh pandemic (*3*). We subjected genomes to a phylogenomic analysis that used Roary version 3.13.0 (https://github.com/sanger-pathogens/Roary), snp-dist version 2.5.1 (https://github.com/sanger-pathogens/snp-sites), and IQtree version 1.6.12 (https://github.com/Cibiv/IQ-TREE).

On the basis of the *V. cholerae* core genome, we determined that the 24 genomes from our study belonged to the Afr9 or Afr12 sublineages, including the clinical and environmental strains (Figure); those 2 sublineages have been associated with cholera outbreaks in countries in West Africa (*3*). The Afr9 genomes showed the wild-type sequence for GyrA and ParC, and the Afr12 genomes showed the S83I (GyrA) and S85L (ParC) mutations. *V. cholerae* strains from Nigeria had been previously characterized with those mutations, which were associated with resistance to nalidixic acid and decreased susceptibility to ciprofloxacin (*5*). The differences between the resistance profile of the Afr9 and Afr12 strains could be observed in the antimicrobial susceptibility profile (Table). Furthermore, we observed other resistance differences, mainly concerning resistance to streptomycin, sulfonamide, trimethoprim/sulfamethoxazole, and chloramphenicol (Table). By analyzing the resistome of the genomes (Appendix), we identified genes associated with resistance to those antimicrobials: *aph*(3′′)-Ib (*str*A) and *aph*(6)-Id (*str*B) (streptomycin), *sul*2 (sulfonamide), *dfr*A1 (trimethoprim/sulfamethoxazole), and *flo*R (chloramphenicol). The genes were located in the integrative and conjugative element STX, which is predominant in genomes of current *V. cholerae* O1 strains, contrasting with 7PET strains from the 1970s (*5*). Of note, VC23, VC62, VC64 (Afr9), and VC105 (Afr12) presented a deletion in the integrative and conjugative element STX region that contained the *str*A/B, *flo*R, and *sul*2 genes, which resulted in differences in the antimicrobial resistance profile between those strains and the others (Table). 

**Figure:**
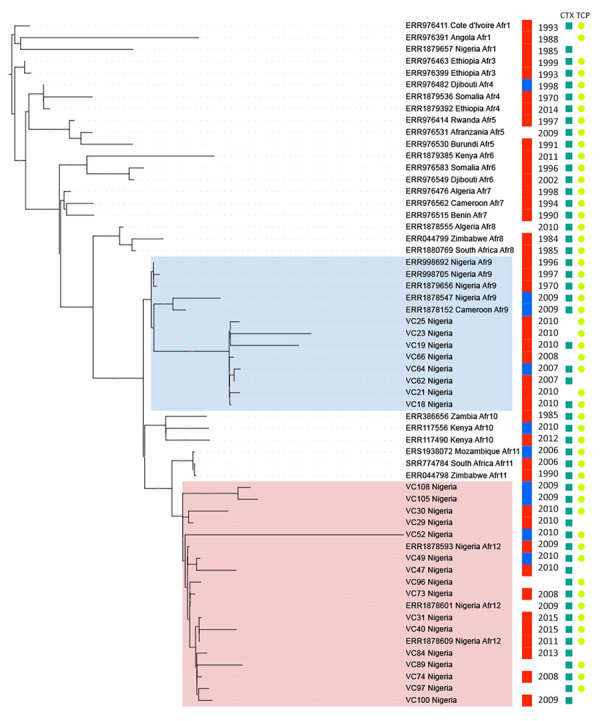
Maximum-likelihood tree of clinical and environmental *Vibrio cholerae* O1 strains, Nigeria, 2008–2015. The best evolutionary model was Kimura 3-parameter plus ascertainment bias correction plus FreeRate model 2, selected by Bayesian information criterion. The highlighted clusters represent genomes from Afr9 (blue) and Afr12 (pink) lineages. To the right of the genome name is information about source of isolation (red, human; blue, environment), year of isolation, presence of *ctx*AB genes (dark green block), and complete TCP cluster (light green circle). Red dots on branches represent >70% bootstrap values. Available GenBank accession numbers are provided. CTX, cholera toxin; TCP, toxin coregulated pilus.

**Table:** Antimicrobial susceptibility profile of environmental and clinical strains of *Vibrio cholerae*, Nigeria*

Strains	Lineage	Source	Isolation date	ICE STX	GEN	STR	NAL	CIP	SUL	SXT	TET	CHL
VC18 VC19 VC21 VC25 VC66	Afr9	Clinical	2008/2010	+	S	R	S	S	R	R	S	S
VC23 VC62	Afr9	Clinical	2007/2010	–	S	I	S	S	S	S	S	S
VC64	Afr9	Water	2007	–	S	I	S	S	S	S	S	S
VC29 VC30 VC31 VC40 VC47 VC73 VC74 VC84 VC100	Afr12	Clinical	2008/2009/2010/2013/2015	+	S	R	R	S	R	R	S	I
VC105	Afr12	Water	2009	–	S	I	R	S	S	S	S	S
VC49 VC52 VC108	Afr12	Water	2009/2010	+	S	R	R	S	R	R	S	I
VC89 VC96 VC97	Afr12	Unknown	Unknown	+	S	R	R	S	R	R	S	I

*Phenotypic resistance was determined according to Clinical and Laboratory Standards Institute breakpoints (*6**,**7*). CHL, chloramphenicol; CIP, ciprofloxacin; GEN, gentamicin; ICE, integrative and conjugative element; NAL, nalidixic acid; STR, streptomycin; SUL, sulfonamide; SXT, trimethoprim/sulfamethoxazole; TET, tetracycline; +, indicates presence of the region with antimicrobial resistance genes in ICE STX; –, indicates absence of the region with antimicrobial resistance genes in ICE STX.

Environmental and clinical genomes were related, particularly observed in 2 pairs of genomes: VC64 (Afr9/environmental/2007) and VC62 (Afr9/clinical/2007), and VC49 (Afr12/environmental/2010) and VC47 (Afr9/clinical/2010) (Figure). All Afr9 and Afr12 environmental genomes from Nigeria harbored the 2 major virulence determinants of epidemic *V. cholerae* O1, the *ctx*AB operon, and the TCP cluster, as well as most clinical genomes (Appendix). Those data represent evidence that strains belonging to the Afr9 and Afr12 epidemic lineages could be recovered from the environment in a West Africa country (Nigeria) and would still harbor the main virulence determinants of *V. cholerae*. A study conducted in East Africa (Tanzania) showed that the Afr10e sublineage, associated with a cholera outbreak in that region, could also be isolated from the environment (fish and water) and, as shown here, also harbored the *ctx*AB operon and the TCP cluster (*8*).

The global initiative for cholera control aims to reduce cholera deaths by 90% by 2030 (*9*). However, despite adoption of cholera elimination measures by many countries, cholera cases in 2023 demonstrated a huge and alarming resurgence across Africa, including Nigeria. The recent resurgence of cholera in some countries in Africa may be associated with climate change (*10*), but evidence of the presence of choleragenic *Vibrio* in the environment reveals the fundamental role of safe drinking water, sanitation, and hygiene in preventing and controlling cholera. Overall, our study highlights the need for continued genomic surveillance considering clinical and environmental *V. cholerae* strains.

Appendix Genomic features of *Vibrio cholerae* isolates analyzed in study of environmental *Vibrio cholerae* strains harboring cholera toxin and *Vibrio* pathogenicity island 1, Nigeria, 2008–2015.
